# Optimizing cohort criteria for multi-country analysis of women experiencing menopause in administrative databases

**DOI:** 10.1186/s12874-026-02830-3

**Published:** 2026-03-28

**Authors:** Victoria Banks, James Brash, Cecilia Caetano, Carina Dinkel-Keuthage, Ronald Herrera, Cécile Janssenswillen, Mariam Saadedine, Nils Schoof, Carsten Moeller

**Affiliations:** 1 Bayer PLC, 400 S Oak Way, Reading, RG2 6AD United Kingdom; 2 IQVIA, 3 Forbury Place, 23 Forbury Road, Reading, RG1 3JH United Kingdom; 3 Bayer Consumer Care AG, Peter Merian-Strasse 84, Basel, 4052 Switzerland; 4 Bayer AG, Pharmaceuticals, Müllerstraße 178, Berlin, 13353 Germany; 5 Department of Obstetrics and Gynecology, George Washington University, Washington, DC, U.S.

**Keywords:** Menopause symptoms, Vasomotor symptoms, Menopause, Real-world data, Phenotype algorithm, Electronic health data, Cohort study, Hormone therapy

## Abstract

**Background:**

Large electronic datasets do not always correctly identify women (defined as individuals whose sex assigned at birth was female, although we acknowledge that biological sex does not always correlate with gender identity) experiencing menopause or their symptoms, making it challenging to generate robust evidence on the burden of menopause. This study aimed to construct and characterize cohorts of women experiencing menopause to explore differences in their identification based on the cohort criteria and to provide recommendations for defining cohorts of individuals experiencing menopause in real-world data.

**Methods:**

This was a retrospective, multi-country cohort study using databases that included a total of ~240 million individuals from five countries: three observational electronic health record databases from France, Germany, and the UK; and two administrative claims databases from Japan and the USA. Demographic, comorbidity, and concomitant medication covariates were used to characterize the study target cohorts at multiple time periods relative to the index event. Counts were calculated for the number of women (as identified in the databases) in each database that matched the target cohort criteria.

**Results:**

Cohorts that included a more specific cohort definition of women aged 40–65 with a probable menopause diagnosis with or without symptom criteria produced age distributions that best matched those expected for women experiencing menopause, along with the highest VMS incidence rates. The prevalence of comorbidities varied between databases and cohorts. However, generally, the most common comorbidities were anxiety, depression, hypertension, hypothyroidism, and osteoarthritis.

**Conclusions:**

The findings provide evidence to support the development of a reproducible definition of menopause and menopause symptoms, which can be used for future analysis of observational healthcare databases.

**Supplementary Information:**

The online version contains supplementary material available at 10.1186/s12874-026-02830-3.

## Background

The menopausal transition typically begins in a woman’s (defined as individuals whose sex assigned at birth was female, although we acknowledge that biological sex does not always correlate with gender identity) late 40s to early 50s. It is characterized by specific symptoms, such as menstrual irregularities, sleep disturbances, and vasomotor symptoms (VMS). Menopause is related to ovarian aging and is established once 12 consecutive months of amenorrhea have passed [1, 2].

Women experiencing menopause can experience a variety of physical and psychological symptoms that impact their quality of life (QoL), work productivity, and daily routine. VMS are often reported as one of the most disruptive symptoms [3, 4], with around one-third of women reporting severe VMS that are associated with interruption of activity as well as impacts on daily tasks and QoL [5–8].

It is important to ensure robust evidence is generated in real-world settings to better characterize symptoms, demographic profiles, as well as the efficacy and safety of specific treatments and interventions. Observational healthcare databases, such as electronic medical records and insurance claim data, offer many advantages for real-world evidence generation, including large sample sizes and accessible longitudinal assessment of patient health information in routine care settings [9, 10]. Yet, studies of menopause utilizing such data are scarce, in part due to the challenges in identifying women experiencing menopause and associated symptoms using clinical data in healthcare databases [11–13]. Women may not be coded under a specific diagnosis of perimenopause or menopause, making identification of the true menopausal population very difficult.

To consistently use healthcare databases for epidemiological research, systematic decisions must be made when determining the appropriate individuals for study inclusion. However, in the few studies published regarding menopause symptoms, the inclusion criteria vary, and there is no standard algorithm for identifying menopausal status or associated symptoms, including VMS [14–20].

This study builds on clinical frameworks for reproductive aging and the menopausal transition, such as the STRAW+10, by developing and testing menopause-related cohort definitions using real-world data in large healthcare databases from five countries with varying medical coding systems. We then investigated how the patient characteristics of each cohort compared to survey-based studies in the literature, where the target population was more accurately identifiable and generalizable [4, 5].

The primary study objective was to understand the differences in demographic and clinical characteristics of women of natural menopausal age when applying differing cohort inclusion and exclusion criteria using administrative databases from different countries. The secondary objective was to understand how different cohort definitions impact the epidemiological estimates of VMS in the cohorts generated for the primary objective. Outputs from this study will provide insights to improve the understanding of the potential sources of variability that have led to diverse estimates of VMS in women experiencing menopause. Results will also inform the development of a reproducible definition of menopause for the analysis of observational healthcare databases.

## Methods

EpiVaSym was a retrospective, multi-country, cohort study using five databases including >240 million individuals from five countries: three observational healthcare electronic health record (EHR) databases from France (Longitudinal Patient Database [LPD]), Germany (Disease Analyzer [DA]), and the United Kingdom (UK Clinical Practice Research Datalink [CPRD] Aurum); and two administrative claims databases from Japan (Japan Claims) and the United States (US MarketScan® Commercial Claims and Encounters Data). For more information regarding the study databases, see Supplementary Table 1.

The use of CPRD Aurum and CPRD GOLD data was approved by the CPRD Research Data Governance Process. The New England Institutional Review Board (IRB) has determined that studies conducted in Marketscan® are exempt from study-specific IRB review, as these studies do not qualify as human subjects research. Based on Ethical Guidelines for Epidemiological Research issued by the Japanese Ministry of Health, Labor and Welfare, ethics approval and informed consent were not applicable for the use of Japan Claims. For France LPD and Germany DA, no patient permission was necessary because all data were deidentified for research purposes.

Databases were standardized to the Observational Medical Outcomes Partnership (OMOP) Common Data Model (CDM), enabling the use of standardized analytics and tools across the network due to a harmonized data structure and terminology system. Specifically, the conversion of ICD-10 codes to SNOMED codes was facilitated using Athena (https://athena.ohdsi.org/), a web-based tool provided and maintained by the Observational Health Data Sciences and Informatics (OHDSI) collaborative (https://www.ohdsi.org/) that provides access to standardized vocabularies and mapping resources within the OMOP CDM. Athena allows users to search for clinical concepts and view their hierarchical and clinical relationships, enabling efficient mapping of ICD-10 codes to their corresponding Systematized Nomenclature of Medicine Clinical Terms (SNOMED CT) identifiers. The mapping process involved utilizing predefined mappings available in Athena, which were validated to ensure clinical accuracy and relevance for research purposes.

Clinical information was represented by codes termed “concept IDs”, which can be collected into “concept sets” to define a clinical topic such as natural menopause. Target cohorts were characterized in terms of covariates for demographics, comorbidities, and medications. Comorbidities were included to explore potential differences in health profiles between cohorts and provide context for interpreting variation across the databases. The full list of comorbidities and medication covariates used to characterize the cohorts is available in Supplementary Table 2. Sleep disturbances were a part of a separate analysis beyond the scope of this paper and were therefore excluded from this analysis.

Concept sets comprised multiple concepts from the standardized OMOP CDM vocabulary and can be combined with specified criteria for the inclusion or exclusion of related concepts found in the vocabulary. ATLAS is a web-based OHDSI tool that facilitates the design and execution of analyses on data standardized to the CDM format. Concept sets can be saved within ATLAS for use in further analyses as components of cohort definitions.

This study was descriptive without formal hypothesis testing. Study size was dependent on the number of women within each database that matched the inclusion criteria for each cohort. All cohorts included individuals recorded as female aged 40 to 65 who had at least 365 days of continuous registration in the database. The index event was defined as the first recorded instance of a menopause diagnosis, menopause-associated symptom, or prescription for a treatment used for menopause (depending on the cohort definition), occurring between January 1, 2010 and the end of the study period. The index event marked the start of follow-up for each individual within the cohort.

Observation within the study database ended at 65 years of age. The end of the study period was defined as the time of the latest available data for each data source. Counts were calculated for the number of women in each database that matched the target cohort criteria. Results were presented as aggregated statistics, and no individuals were identifiable.

Cohorts were defined based on three factors: 1) the likelihood of the cohort definition identifying menopause; 2) the inclusion or exclusion of an explicit descriptor of menopause; and 3) the inclusion or exclusion of a prescription for treatment used for menopause-associated symptoms.

Table 1 summarizes the cohorts that were built. Cohort 1 included all women aged between 40 and 65 with at least 365 days of continuous observation in the database and no other inclusion criteria. For Cohorts 2–5, inclusion criteria were as per Cohort 1, with the following additional criteria; Cohort 2: a diagnosis of menopause, Cohort 3: a diagnosis of menopause or a menopause symptom, Cohort 4: one or more condition, observation, or procedure for a menopause diagnosis or menopause symptom, or a prescription for a hormonal or non-hormonal treatment indicated for menopause-associated symptoms, Cohort 5: one or more prescription for menopause treatment (including estradiol, conjugated estrogens, estrogen depot, tibolone, estriol, estetrol, hormone combinations, progestogens, topical estrogens, clonidine, serotonin-norepinephrine reuptake inhibitors [SNRIs]/selective serotonin reuptake inhibitors [SSRIs]). Full details of the concept IDs used, including those for menopause symptoms and treatments, are provided in the Concept IDs spreadsheet included in the supplementary material.

**Table 1:** Definitions, inclusion and exclusion criteria for menopause cohorts

**Target cohorts**		**Inclusion criteria**^**a**^						**Exclusion criteria**^**b**^
		**Aged 40–<65 years**	**Menopause diagnosis**		**Symptom of menopause**		**Hormonal or non-hormonal treatment**	**Prior hormonal or non-hormonal treatment**
			*Probable*	*Possible*	*Menopause descriptor*	*No menopause descriptor*		
Cohort 1) Women of natural menopausal age	C1	X						
Cohort 2) Women of natural menopausal age with a diagnosis of menopause	C2a	X	X	X				
	C2b	X	X	X				X
	C2c	X	X					
	C2d	X	X					X
Cohort 3) Women of natural menopausal age with a diagnosis of menopause or menopause-specific symptom	C3a	X	X	X	X	X		
	C3b	X	X	X	X	X		X
	C3c	X	X		X			
	C3d	X	X		X			X
Cohort 4) Women of natural menopausal age with a diagnosis of menopause, menopause symptom, or prescribed treatment used for menopause	C4a	X	X	X	X	X	X	
	C4b	X	X		X		X	
Cohort 5) Women of natural menopausal age with a prescribed treatment used for menopause	C5	X					X	

^a^All cohorts specified at least 365 days of continuous observation in the database prior to the index event

^b^All menopause cohorts excluded patients with prior bilateral oophorectomy (possible) and/or radical hysterectomy (possible) before index event and/or previous exposure to endocrine adjuvant therapy

Exclusion criteria for all cohorts included procedure or observation of a record of bilateral oophorectomy or radical hysterectomy at any time on or before the index event or exposure to endocrine adjuvant therapy (tamoxifen, gonadotropin-releasing hormone analogs, or aromatase inhibitors) in the 12 months prior to or on the index event. For Cohorts 1, 2, & 4, those with a record of exposure to a hormonal (including estradiol, conjugated estrogens, estrogen depot, tibolone, estriol, estetrol, hormone combinations, progestogens, topical estrogens) or non-hormonal treatment (including clonidine, SSRIs/SNRIs, paroxetine) in the 12 months prior to the index event were also excluded.

As the clinical codes in the databases may not explicitly reference menopause, cohorts were designed both with and without a “menopause descriptor” (Supplementary Tables 3 and 4). The codes used in the "probable" cohort definitions were more likely to identify women experiencing menopause, or menopause with VMS in the VMS outcome cohort, while the “possible” cohort definitions included criteria that may or may not relate to menopause or menopause with VMS (for example, ovarian failure; Supplementary Table 5, or abnormal vasomotor function; Supplementary Table 6).

### Analyses & statistics

All analyses were conducted using tools developed by the OHDSI network, a collaborative that provides open-source software for healthcare database research. The OHDSI tools have undergone extensive validation and are actively maintained to ensure reliability and reproducibility [21]. Statistical analyses were implemented using the R package VMSChar, which builds on the OHDSI Cohort Diagnostics framework [22] and is designed to evaluate patient cohorts based on standardized health data.

We conducted descriptive analyses to characterize each cohort in terms of demographics, comorbidities, and medication use. Demographic and clinical information were identified and extracted from our health data by employing the FeatureExtraction R package. This information was then summarized, with non-normally distributed continuous variables presented as medians and categorical variables as proportions.

The VMS incidence rates for each cohort and database were calculated per 1,000 person-years by dividing the total number of new VMS cases in the cohort during the study period by the cohort's total person-time at risk and multiplying by 1,000. Person-time refers to the cumulative time (years) that women remained in the cohort without a prior VMS diagnosis, from the index event until the first occurrence of VMS, end of the observation period, or age 65. VMS incidence findings for each cohort were further categorized into either “probable” or “possible” based on the likelihood that the recorded symptom was related to menopause.

In addition, the PheValuator tool was used to assess the accuracy of the algorithms used to define VMS cases [23, 24]. By generating a reference set through predictive modeling, this tool helps determine the probability that women identified by our algorithms truly have VMS.

Further details of the statistical analyses and software packages used are described in Supplementary Methods.

## Results

### Cohort counts of women of menopausal age

In each of the five study databases, there were between 3,746,801 and 16,076,243 women aged 40–65 with no other inclusion criteria. In CPRD Aurum (UK), cohort sizes were similar for women aged 40–65 years with a possible or probable menopause diagnosis without further inclusion or exclusion criteria (C2a: 5.2%; C2c: 4.2%) (Table 2). Adding the exclusion criterion of prior treatment with hormonal therapy (HT) or non-HT reduced the cohort sizes of these groups by approximately half (C2a to C2b: 5.2% to 3.0%; C2c to C2d: 4.2% to 2.3%). On the other hand, of all women aged 40–65 years, adding the inclusion criterion of first HT or non-HT prescription to cohorts with a menopause diagnosis or symptom significantly increased the cohort sizes (C4a: 27.5%; C4b: 22.5%). Having the inclusion criterion of HT or non-HT treatment alone resulted in ~17.5% of all women aged 40–65 years being included in the cohort.

**Table 2:** Menopause cohort counts for each of the five study healthcare databases

	**C1**	**C2a**	**C2b**	**C2c**	**C2d**	**C3a**	**C3b**	**C3c**	**C3d**	**C4a**	**C4b**	**C5**
CPRD Aurum (UK)	3,752,026 (100%)	193,411 (5.2%)	111,803 (3.0%)	159,124 (4.2%)	86,661 (2.3%)	624,050 (16.6%)	431,648 (11.5%)	386,937 (10.3%)	222,458 (5.9%)	1,030,019 (27.5%)	847,632 (22.6%)	656,034 (17.5%)
Germany DA	3,746,801 (100%)	158,779 (4.2%)	120,029 (3.2%)	87,802 (2.3%)	61,510 (1.6%)	424,267 (11.3%)	367,630 (9.8%)	102,592 (2.7%)	73,696 (2.0%)	567,292 (15.1%)	304,354 (8.1%)	240,575 (6.4%)
France LPD	1,704,721 (100%)	65,951 (3.9%)	40,007 (2.4%)	488 (0.0%)	265 (0.0%)	287,684 (16.9%)	229,794 (13.5%)	3,188 (0.2%)	2,129 (0.1%)	412,179 (24.2%)	201,013 (11.8%)	198,898 (11.7%)
Japan Claims	700,712 (100%)	83,914 (12.0%)	74,534 (10.6%)	50,436 (7.2%)	42,661 (6.1%)	212,403 (30.3%)	200,627 (28.6%)	53,746 (7.7%)	45,556 (6.5%)	251,873 (35.9%)	115,918 (16.5%)	84,817 (12.1%)
US Market-scan®	16,076,243 (100%)	2,211,929 (13.8%)	1,379,060 (8.6%)	1,993,314 (12.4%)	1,213,209 (7.5%)	3,977,099 (24.7%)	2,868,857 (17.8%)	2,151,346 (13.4%)	1,328,079 (8.3%)	5,516,137 (34.3%)	3,979,274 (24.8%)	2,532,314 (15.8%)

*C1:* women of natural menopausal age (aged 40–65); *C2a:* women aged 40–65 with a possible menopause diagnosis; *C2b:* women aged 40–65 with a possible menopause diagnosis and no prior HT or non-HT treatment; *C2c:* women aged 40–65 with a probable menopause diagnosis; *C2d:* women aged 40–65 with a probable menopause diagnosis and no prior HT or non-HT treatment; *C3a:* women aged 40–65 with a possible menopause diagnosis or symptom with or without menopause descriptor; *C3b:* women aged 40–65 with a possible menopause diagnosis or symptom with or without menopause descriptor and no prior HT or non-HT treatment; *C3c:* women aged 40–65 with a probable menopause diagnosis or symptom with menopause descriptor; *C3d:* women aged 40–65 with a probable menopause diagnosis or symptom with menopause descriptor and no prior HT or non-HT treatment; *C4a:* women aged 40–65 with a possible menopause diagnosis or symptom with or without menopause descriptor or a HT/non-HT treatment; *C4b:* woman aged 40–65 with a probable menopause diagnosis or symptom with menopause descriptor or a HT/non-HT treatment; *C5:* women aged 40–65 with a HT/non-HT treatment

*CPRD *Clinical Practice Research Datalink, *DA *Disease Analyzer, *HT *Hormone therapy, *LPD *Longitudinal Patient Database, *UK *United Kingdom, *US *United States

Similar trends to CPRD Aurum were generally seen in the Germany DA and France LPD in terms of the impact of different criteria on the percentage of women aged 40–65 entering each cohort, with some differences. Cohorts requiring a probable menopause diagnosis or symptoms with menopause descriptor were much smaller when compared with those requiring a possible menopause diagnosis or symptoms with/without descriptor (Germany DA: 11.3% [C3a; possible diagnosis] vs. 2.7% [C3c; probable diagnosis]; France LPD: 16.9% [C3a; possible diagnosis] vs. 0.2% [C3c; probable diagnosis]).

In the Germany DA, adding the exclusion criterion of prior HT or non-HT use (most commonly prescribed for VMS) had a much smaller impact than that observed in CPRD Aurum (UK), with each of the relevant cohort definitions reduced by <1.5% of all women aged 40–65 years. Adding first prescription of HT or non-HT as an inclusion criterion in the Germany DA increased the cohort sizes of C4a (possible menopause diagnosis or symptoms with/without descriptor or HT/non-HT) and C4b (probable menopause diagnosis or symptoms with descriptor or HT/non-HT) to approximately 15% and 8%, respectively. Similar trends were also seen in the France LPD, although definitions for probable menopause diagnosis or symptoms with menopause descriptor did not seem feasible to execute in this data source as all related cohorts comprised <1% of women aged 40–65 years.

Marketscan® (US) and Japan Claims cohorts generally included higher proportions of women aged 40–65 than any of the European databases. The differences between cohorts with a possible menopause diagnosis (C2a, C2b, C3a, C3b, C4a) compared with cohorts requiring a probable menopause diagnosis (C2c, C2d, C3c, C3d, C4b) were the greatest in Japan Claims (C2a [12.0%] vs. C2c [7.2%]; C2b [10.6%] vs. C2d [6.1%]; C3a [30.3%] vs. C3c [7.7%]; C3b [28.6%] vs. C3d [6.5%]; C4a [35.9%] vs. C4c [16.5%) and Marketscan® (C2a [13.8%] vs. C2c [12.4%]; C2b [8.6%] vs. C2d [7.5%]; C3a [24.7%] vs. C3c [13.4%]; C3b [17.8%] vs. C3d [8.3%]; C4a [34.3%] vs. C4c [24.8%]). Unlike France LPD, the approach regarding probable menopause diagnosis still seemed feasible for defining a cohort of women experiencing natural menopause (relevant cohort range 7.5% to 24.8% in Marketscan®; range 6.1% to 16.5% in Japan Claims).

### Age distribution of menopause cohorts

**Fig. 1:**
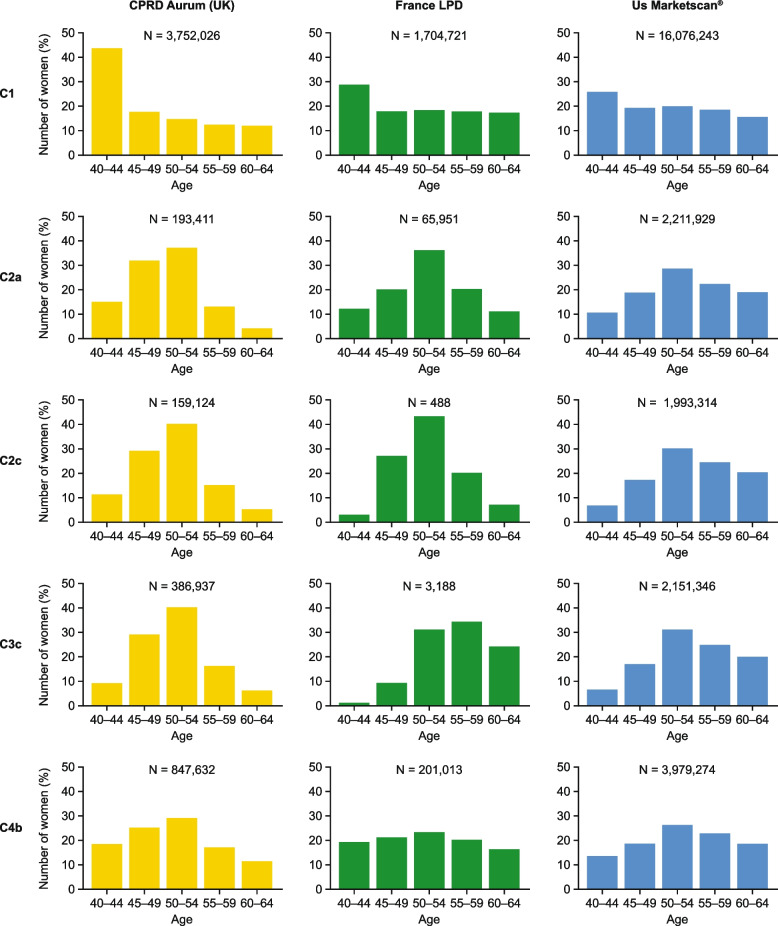
Age distribution of menopause cohorts C1, C2a, C2c, C3a, and C4b for CPRD Aurum UK, France LPD, US Marketscan®. *C1:* women of natural menopausal age (aged 40–65); *C2a:* women aged 40–65 with a possible menopause diagnosis; *C2c:* women aged 40–65 with a probable menopause diagnosis; *C3c:* women aged 40–65 with a probable menopause diagnosis or symptom with menopause descriptor; *C4b:* women aged 40–65 with a probable menopause diagnosis or symptom with menopause descriptor or a HT/non-HT treatment. CPRD, Clinical Practice Research Datalink; LPD, Longitudinal Patient Database; UK, United Kingdom; US, United States

Figure 1 shows the age distributions for cohorts C1, C2a, C2c, C3c, and C4b for CPRD Aurum, France LPD, and US Marketscan® (in 5-year age bands), with the age distribution for each of the menopause cohorts for all five data sources shown in Supplementary Figure 1. For each database, cohort C1 (women of natural menopausal age) was skewed towards a higher percentage of women aged 40–44, with a relatively flat distribution across other ages (45–65), except for Japan Claims, where a higher proportion of women were aged 40–54 than in the other data sources.

In CPRD Aurum (UK), Germany DA, and Japan Claims, cohorts that included a probable menopause diagnosis (with/without symptom criteria [C2c, C2d, C3c, C3d]) had the expected unimodal age distribution. Whilst this was also the case for cohorts with a probable menopause diagnosis in the France LPD (C2c, C2d), the addition of menopause symptoms with a descriptor (C3c, C3d) saw a much more right-skewed distribution towards a greater proportion of women aged 50–65 within the cohort. In the US Marketscan® database, the age distributions for cohorts with a probable menopause diagnosis, with/without symptom criteria, were slightly skewed towards greater numbers of older women.

The addition of the criteria for menopause symptoms with/without a descriptor (C2a and C2b) resulted in a flatter distribution for all data sources. This was also the case for cohorts C4a and C4b, where symptoms and treatment use were inclusion criteria along with a menopause descriptor.

### Comorbidities

**Fig. 2:**
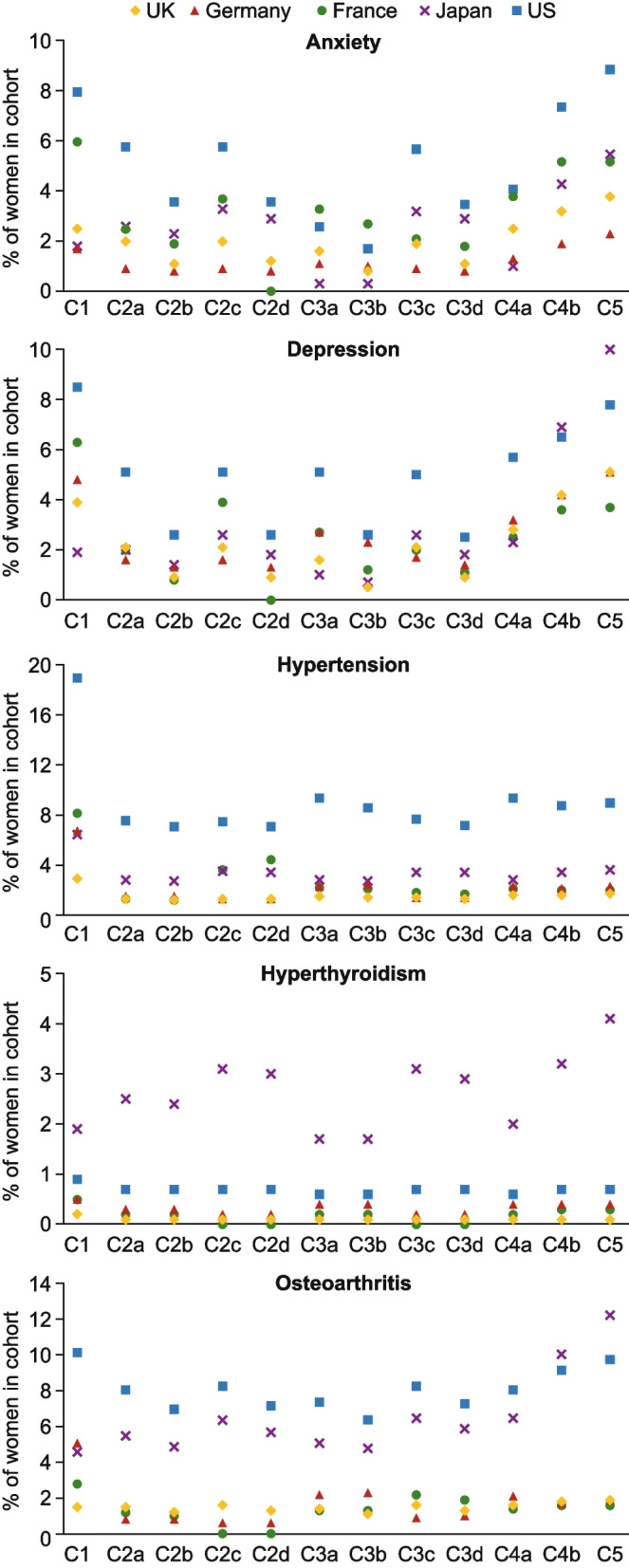
Comorbidities in the menopause cohorts (12 months before index), including (**a**) anxiety, (**b**) depression, (**c**) hypertension, (**d**) hyperthyroidism, (**e**) osteoarthritis. *C1:* women of natural menopausal age (aged 40–65); *C2a:* women aged 40–65 with a possible menopause diagnosis; *C2b:* women aged 40–65 with a possible menopause diagnosis and no prior HT or non-HT treatment; *C2c:* women aged 40–65 with a probable menopause diagnosis; *C2d:* women aged 40–65 with a probable menopause diagnosis and no prior HT or non-HT treatment; *C3a:* women aged 40–65 with a possible menopause diagnosis or symptom with or without menopause descriptor; *C3b:* women aged 40–65 with a possible menopause diagnosis or symptom with or without menopause descriptor and no prior HT or non-HT treatment; *C3c:* women aged 40–65 with a probable menopause diagnosis or symptom with menopause descriptor; *C3d:* women aged 40–65 with a probable menopause diagnosis or symptom with menopause descriptor and no prior HT or non-HT treatment; *C4a:* women aged 40–65 with a possible menopause diagnosis or symptom with or without menopause descriptor or a HT/non-HT treatment; *C4b:* women aged 40–65 with a probable menopause diagnosis or symptom with menopause descriptor or a HT/non-HT treatment; *C5:* women aged 40–65 with a HT/non-HT treatment. HT, hormone therapy; UK, United Kingdom; US, United States

Generally, the most common of comorbidities (excluding VMS and sleep disturbances) were anxiety (Fig. 2a), depression (Fig. 2b), hypertension (Fig. 2c), hypothyroidism (Fig. 2d), and osteoarthritis (Fig. 2e). For four of the five top comorbidities, a higher percentage of women with each comorbidity was observed for all cohorts in Marketscan® (US) compared with Japan Claims and the European databases, with the exception of C4b for depression and osteoarthritis, where the percentages in Japan Claims were slightly higher.

The prevalence of hyperthyroidism was similarly low for all cohorts in all databases except for Japan Claims (Fig. 2d), where the percentage of women with hyperthyroidism was consistently higher for each cohort. The prevalence of osteoarthritis was considerably lower across all cohorts in the European databases than in Japan Claims or Marketscan® (US) (Fig. 2e).

### Menopause-associated VMS

**Fig. 3:**
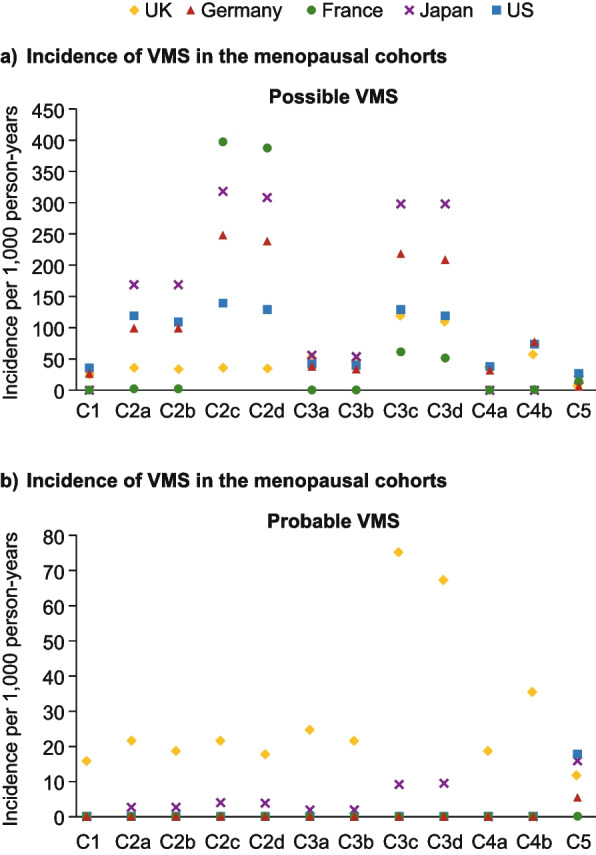
Incidence of (**a**) possible VMS and (**b**) probable VMS in the menopause cohorts. *C1:* women of natural menopausal age (aged 40–65); *C2a:* women aged 40–65 with a possible menopause diagnosis; *C2b:* women aged 40–65 with a possible menopause diagnosis and no prior HT or non-HT treatment; *C2c:* women aged 40–65 with a probable menopause diagnosis; *C2d:* women aged 40–65 with a probable menopause diagnosis and no prior HT or non-HT treatment; *C3a:* women aged 40–65 with a possible menopause diagnosis or symptom with or without menopause descriptor; *C3b:* women aged 40–65 with a possible menopause diagnosis or symptom with or without menopause descriptor and no prior HT or non-HT treatment; *C3c:* women aged 40–65 with a probable menopause diagnosis or symptom with menopause descriptor; *C3d:* women aged 40–65 with a probable menopause diagnosis or symptom with menopause descriptor and no prior HT or non-HT treatment; *C4a:* women aged 40–65 with a possible menopause diagnosis or symptom with or without menopause descriptor or a HT/non-HT treatment; *C4b:* women aged 40–65 with a probable menopause diagnosis or symptom with menopause descriptor or a HT/non-HT treatment; *C5:* women aged 40–65 with a HT/non-HT treatment. HT, hormone therapy; UK, United Kingdom; US, United States; VMS, vasomotor symptoms

Figure 3 shows the incidence per 1,000 person-years of *possible* (Fig. 3a) and *probable* (Fig. 3b) VMS for each cohort and data source. The overall incidence was generally low for each data point, ranging from 0 to approximately 400 per 1,000 person-years.

For *possible* VMS (Fig. 3a), across most countries, C2c and C2d showed the highest rates of VMS diagnoses. This was greatest in C2c for all data sources, approximately doubling that seen in C2a and C2b, except for CPRD Aurum (UK), where the highest VMS rate was seen in C3c. The second highest incidence rates were seen in C3c and C3d for all databases except for CRPD Aurum (UK), where C4b had the second highest possible VMS incidence of all cohorts.

In cohorts C3a and C3b, incidence rates were considerably lower than those seen in C2a, C2c, C2d, C3c, or C3d; ranging from 50 to 300 per 1,000 person-years across all data sources.

Adding the prescription of HT or non-HT treatment as an inclusion criterion (C4a, C4b) resulted in lower incidence rates of *possible* VMS than those seen in cohorts without a treatment-related criterion (Fig. 3a). France LPD appeared to be an anomaly, with *possible* VMS rates of less than 65 per 1,000 person-years in all cohorts except C2c and C2d. Conversely, it had the highest incidence rates in cohorts C2c and C2d compared with all other data sources; of note, however, sample sizes were very low (<0.1% of all women aged 40–65). The lowest rates for *possible* VMS were generally seen in cohorts C1 and C5, which included women aged 40–65 with no requirement for diagnosis/symptom/treatment and no requirement for HT/non-HT treatment, respectively.

For *probable* VMS (Fig. 3b), incidence rates for Germany DA, France LPD, and US Marketscan® were zero in all cohorts except C1, and similarly low for Japan Claims (less than 10 per 1,000 person-years in all cohorts except C1, zero in C4a and C4b). Although rates in cohort C1 were above zero for all data sources, they were consistently low at less than 20 per 1,000 person-years. For CRPD Aurum (UK), C3c, C3d, and C4b had the highest *probable* VMS incidence rates at 76, 68, and 36 per 1,000 person-years, respectively.

## Discussion

This study aimed to understand the impact of database variations and the use of differing cohort criteria on the demographics and clinical characteristics of women of menopausal age. Knowledge of these possible sources of variability has the potential to inform the development of a reproducible definition of menopause and its associated symptoms, which could subsequently be used to analyze healthcare databases worldwide.

Women aged 50–54 constituted the largest proportion of our menopause cohorts, aligning with the age of natural menopause [3–5, 25]. Overall, cohorts that included a probable menopause diagnosis with or without symptom criteria (C2c, C3c) produced the age distributions that best matched those expected for women experiencing menopause, as well as the highest VMS incidence rates of the cohorts.

The cohorts included in this study encompassed both broad and specific approaches to identify women for inclusion in menopause studies. Despite some published studies [14] using a broad definition to identify women experiencing menopause in real-world secondary data, this approach is more susceptible to misclassifying women as menopausal. We found that adding menopause-related symptom criteria resulted in an increased inclusion of women aged 40–65 in the cohort for most databases. Thus, the use of more specific criteria contributed to shifts in the demographic and clinical characteristics toward distributions that better align with the published literature regarding menopausal populations [3, 7], except for France LPD, where these more specific criteria led to a noticeable decrease in sample size. The addition of a prior treatment used for menopause as an inclusion criterion reduced sample size without a notable gain in specificity and did not appear to improve the cohort definition.

Comorbidities were included to provide a broader understanding of the clinical context in which menopause is recorded. The prevalence of comorbidities was found to vary between the databases and cohorts in this study, and more women with comorbidities were observed in the US and Japan databases. In addition, for France LPD and, to a lesser extent, Germany DA, the availability of codes representing probable menopause was limited, likely reflecting regional/country variations in coding. The observed differences in demographic and clinical characteristics between databases likely reflect a combination of factors, including those associated with healthcare system structure, data capture methods, and coding practices, as well as biological, environmental, and healthcare-related factors. Regarding differences in clinical coding, in claims-based systems such as those in the US and Japan, where reimbursement relies on the accuracy of the clinical coding in the healthcare setting, more detailed clinical coding may be incentivized. However, this is not a universal feature of all healthcare systems.

Menopause symptoms, including VMS, were applied as inclusion criteria for cohorts C3a–d, 4a, and 4b, only. Thus, it can be expected that the VMS incidence rates for these cohorts will have been raised beyond what would otherwise have been reported. However, the results for these cohorts remained within a similar range of VMS incidence to what was found in the other cohorts. Cohorts 2c and 2 d showed the highest VMS incidence rates despite not having VMS (menopause symptoms) as cohort inclusion criteria.

When compared with previous studies [14–20], estimates of VMS were low across all our study cohorts and countries. While this could be expected due to limitations associated with symptom records in secondary databases, there was variation in VMS estimates and the age distributions between cohort definitions, with differing degrees of variation between data sources. The use of SNOMED coding by CPRD Aurum (UK) rather than the ICD-9/10 coding used by the other data sources explored in this study likely contributed to this variation. SNOMED provides a more granular coding system than ICD coding, resulting in more specific coding for probable VMS in CPRD Aurum (UK) compared to the data sources using ICD coding.

Although variation in coding systems may be a driver for the incidence rates of probable VMS observed in this study, it is also important to note that women’s experiences of menopause can vary between countries, which could be evident when comparing healthcare databases from different geographical regions [3, 26]. This may account for some of the variations we saw between databases, such as the differing age distributions for Japan Claims compared to those for the other databases.

Overall, the phenotyping algorithm with a more specific cohort definition of “women aged 40–65 with a probable menopause diagnosis or symptoms with menopause descriptor” was preferable for defining a cohort of women experiencing menopause in real-world data. When designing cohorts, we make several recommendations informed by this study:

- Researchers should consider healthcare systems and coding practices when defining phenotype algorithms for identifying cohorts.
- Explore the prevalence of codes within a database and consider cultural factors that may be important before finalizing the algorithm.
- Employ feasibility counts for concept sets within each database before finalizing the phenotyping algorithm.
- Consider whether the algorithm is suitable for different healthcare settings and for which it has been optimized.
- Determine whether a broad or narrow approach is the most appropriate, considering whether to identify a larger sample size “possibly” with the condition of interest or a smaller sample size that minimizes misclassification into the cohort.
- Use medical expertise to develop concept sets that meet the specific needs of the research question.
- Consider the relevance of each criterion in the context of the study objectives and other inclusion/exclusion criteria to ensure that each criterion improves the robustness of the phenotyping algorithm and is not superfluous.

### Study strengths & limitations

Study strengths included the use of up-to-date real-world data that reflected routine clinical menopause care and the development of multiple target cohorts based on a range of definitions used. Other strengths were the simultaneous, standardized, and directly comparable multi-country cohorts created, as well as the inclusion of countries with different social and cultural bases. These allowed for the observation of differences across countries in real-world coding patterns and clinical practice, along with the inclusion of different healthcare systems and database types.

In general, the limitations of this study were those commonly found in studies adopting administrative and routine medical data for research purposes. Selection bias is a known problem in retrospective EHR databases from healthcare providers and varies between data sources. Data that are of interest but not essential for patient treatment may not be recorded due to differing physician perception, motivation, and clinical practice, as well as under-reporting of symptoms by patients and physicians.

As many women do not seek treatment for menopause symptoms [26] or choose to manage their symptoms using over-the-counter products [27], this study was biased towards women who actively sought medical treatment for their symptoms. Thus, the data included a subset of women whose symptoms were more likely to be moderate to severe. It is also possible that, while menopause may not be the primary reason for women to visit their healthcare provider, the visit may still represent an opportunity to seek advice and treatment for milder menopause symptoms. A proportion of women who are menopausal but do not experience or report symptoms will always be under-identified.

Due to the number and variety of possible symptoms, menopause can be difficult to identify. Therefore, clinicians' first reports of menopause may not be reflective of the true age of onset or incidence of menopause symptoms. As VMS can last 7–10 years postmenopause, healthcare providers are unlikely to code the presence of VMS or other menopause symptoms at each healthcare visit in a consistent fashion, which may result in the underestimation of VMS prevalence. Due to this, the results reported here focused on incidence rates of VMS in newly diagnosed women experiencing menopause only.

The use of menopause symptoms, including VMS, as inclusion criteria for some but not all of the cohorts might have influenced the potential accuracy of the VMS incidence findings and limited the conclusions that can be drawn by comparing these findings with cohorts where VMS were not applied as an inclusion criterion.

It is important to note that comparison between our estimates of menopause and those in the existing literature was limited due to methodological differences between previous studies and this work. For example, the SWAN study [5] enrolled participants aged 42 to 52 years who had menstruated in the previous three months, thereby excluding individuals who experienced early or late menopause. As such, comparisons between our broader age-based cohorts (40–65 years) and SWAN should be approached with caution.

It is also worth noting that the absence of a specific diagnosis, procedure, or drug code is often interpreted as the absence of the disease, procedure, or medication in medical records. This practice could yield a high positive predictive value and compromise sensitivity, leading to potential misclassification of women experiencing menopause. Furthermore, a lack of specificity in coding, whereby menopause is coded but not the specific associated symptoms, may contribute to the underestimation of menopause symptom prevalence. Considering these limitations, medical expertise was used to inform the design of our study cohorts to help ensure that symptoms were identified where coded.

Due to a limitation of the ATLAS platform, where censoring cannot be done on a demographic, individuals were censored at the end of observation, which could have been after the age of 65. Based on a feasibility assessment indicating a mean follow-up time of less than 4 years in the UK and US databases, this limitation is expected to have a nominal impact on study results. However, it should be considered when interpreting the findings in the older age groups.

This work was based on real-world data reflecting routine clinical care in the study countries. Consequently, the results reflected differences between countries over the study period in clinical practice, VMS diagnosis, treatment guidelines, healthcare system and reimbursement, as well as social and cultural aspects.

## Conclusions

Large electronic datasets do not always correctly identify women experiencing menopause or their symptoms, making it challenging to generate robust evidence on the burden of menopause. Our recommendations for defining phenotype algorithms for study cohorts include considering local variations in coding systems, using medical expertise to develop concept sets, employing feasibility counts for concept sets, and taking into account the relevance of each criterion within the context of the study objectives. Researchers need to look beyond the definition of women experiencing menopause as those aged 40–65 and consider diagnosis, procedure, and symptom codes, such as those described in this study, for possible and probable menopause.

Improvements in clinical coding vocabulary as well as physicians' recording of menopause and its symptoms are urgently needed. Until then, the absence of a gold-standard definition and a lack of coding uniformity mean that the epidemiology of menopause symptoms will continue to be underestimated and biased in real-world secondary databases. We hope that insights from this study highlight the need for and support the development of a reproducible definition of menopause and menopause symptoms, which can be used for future analysis of observational healthcare databases.

## Supplementary Information

Supplementary Material 1: Supplementary Methods. Supplementary Table 1: Description of data sources. Supplementary Table 2: Covariates and time restrictions. Supplementary Table 3: Natural menopause diagnosis concept IDs. Supplementary Table 4: Menopause descriptor concept IDs. Supplementary Table 5: Symptoms without menopause descriptor concept IDs. Supplementary Table 6: Codes used to identify VMS-related records. Supplementary Figure 1: Age distribution of menopause cohorts.

## Data Availability

The data that support the findings of this study are available from IBM Marketscan US, Clinical Practice Research Datalink, IQVIA, and Optum Clinformatics® Data Mart, but restrictions apply to the availability of these data, which were used under license for the current study, and so are not publicly available.

## Abbreviations

CDM: Common Data Model
CPRD: Clinical Practice Research Datalink
DA: Disease Analyzer
EHR: Electronic health record
HT: Hormone therapy
LPD: Longitudinal Patient Database
OHDSI: Observational Health Data Sciences and Informatics
OMOP: Observational Medical Outcomes Partnership
QoL: Quality of life
NK: Neurokinin
SNRI: Serotonin norepinephrine reuptake inhibitor
SSRI: Selective serotonin reuptake inhibitor
SWAN: Study of Women’s Health Across the Nation
UK: United Kingdom
US: United States
VMS: Vasomotor symptoms
