# Supplementary material for: Optimizing cohort criteria for multi-country analysis of women experiencing menopause in administrative databases
Source: BMC Med Res Methodol. 2026 Mar 28;26:79. doi: 10.1186/s12874-026-02830-3 (PMC13063593; doi:10.1186/s12874-026-02830-3)
Supplement: Supplementary file 1 — Supplementary Material 1: Supplementary Methods. Supplementary Table 1: Description of data sources. Supplementary Table 2: Covariates and time restrictions. Supplementary Table 3: Natural menopause diagnosis concept IDs. Supplementary Table 4: Menopause descriptor concept IDs. Supplementary Table 5: Symptoms without menopause descriptor concept IDs. Supplementary Table 6: Codes used to identify VMS-related records. Supplementary Figure 1: Age distribution of menopause cohorts. [file 12874_2026_2830_MOESM1_ESM.docx]

Supplementary Material

**Supplementary Methods**

To describe the baseline characteristics of patients in each cohort, predefined covariates that capture demographic and clinical information were extracted using a Structured Query Language script that had been optimized for performance, based on the principles of the FeatureExtraction R package [1]. FeatureExtraction is a tool that generates patient-level features (covariates) from observational health data that has been standardized to the OMOP CDM. In addition to the predefined covariates of age, sex, periods during which the patient had a specific diagnosis, condition, or medication, custom variables tailored to each cohort were created using clinical concepts from the OMOP Standardized Vocabulary.

When the FeatureExtraction process was executed, the VMSChar package generated a structured dataset (data frame) that included each patient’s age and sex. Baseline disease characteristics at the index date (the date of cohort entry) were summarized using medians for continuous variables that did not follow a normal distribution, and proportions for categorical variables.

The cohort entry event was the first recorded occurrence of a VMS-related condition or observation in a patient’s history occurring between January 1, 2010 and the end of the study period. The codes used to identify VMS-related records were selected based on clinical relevance and included terms such as ‘menopausal flushing’ (198436008), ‘postmenopausal flushing’ (403389006), ‘night sweats’ (42984000), and ‘hot sweats’ (224962007) (a full list is provided in Supplementary Table 6). For cohorts where VMS was already an inclusion criterion (Cohorts 3a–d, 4a, and 4b), the presence of VMS was determined based on the clinical codes used to define the cohort.

A tool called PheValuator was used to evaluate the accuracy of the algorithms used to define VMS cases [2,3]. PheValuator estimates the probability that patients identified by a given algorithm truly have the condition of interest by generating a reference set using predictive modeling. The models were developed using the PatientLevelPrediction R package, which applies machine learning methods to estimate individual-level probabilities of having a specific health outcome. PheValuator is part of the Health Analytics Data to Evidence Suite, previously known as the OHDSI Methods Library.

[1] Schuemie MJ, Suchard MA, Ryan PB, Reps J, Sena A, Inberg G. FeatureExtraction. 2022. Available from: https://ohdsi.github.io/FeatureExtraction/ Accessed July 2024.

[2] Swerdel JN, Hripcsak G, Ryan PB. PheValuator: development and evaluation of a phenotype algorithm evaluator. J Biomed Inform. 2019;97:103258.

[3] Swerdel JN, Schuemie M, Murray G, Ryan PB. PheValuator 2.0: methodological improvements for the PheValuator approach to semi-automated phenotype algorithm evaluation. J Biomed Inform. 2022;135:104177.

**Supplementary Table 1: Description of data sources**

| **Data source** | **Source population** | **Sample size (millions)** | **Data type** | **Longitudinal history** | **Percentage population covered** |
| --- | --- | --- | --- | --- | --- |
| Germany DA | Ambulatory | 38.5 | Electronic health records | 1992 onwards | 46.0% |
| France LPD | Ambulatory | 17.9 | Electronic health records | 1994 onwards | 27.0% |
| UK CPRD Aurum | Ambulatory | 13.3 | Electronic health records | 2021 onwards | 19.4% |
| US IBM MarketScan® | Closed claims | 164.8 | Claims data | 2002 onwards | NA |
| Japan Claims | Closed claims | 3.5 | Claims data | 2013 onwards | 2.8% |

CPRD, Clinical Patient Research Datalink; DA, Disease Analyzer; IBM, International Business Machines; LPD, Longitudinal Patient Database; NA, not available; UK, United Kingdom; US, United States.

Supplementary Table 2: Covariates and time restrictions

| **Covariates** | **Time restriction** |
| --- | --- |
| **Demographics** | |
| Age as a continuous variable | At index date |
| Age in 5-year categories | At index date |
| Charlson Comorbidity Index (CCI) (0, 1, ≥2) | At index date |
| **Comorbidities** | |
| Alopecia | No restraint |
| Anxiety | No restraint |
| Breast cancer | No restraint |
| Depression | No restraint |
| Depression w/o bipolar disorder | No restraint |
| Gynecological cancer (ex. cervical cancer) | No restraint |
| Hypertension  Hyperthyroidism  Hypothyroidism | No restraint  No restraint  No restraint |
| Malaise or fatigue | No restraint |
| Myocardial infarction | No restraint |
| Osteoarthritis | No restraint |
| Osteoporosis | No restraint |
| Type 2 diabetes (no Type 1 diabetes) | No restraint |
| Stroke (ischemic or hemorrhagic) | No restraint |
| Venous thromboembolism | No restraint |
| **Medications** | |
| Antidepressants | No restraint |
| Antihistamines | No restraint |
| Aromatase inhibitors | No restraint |
| Benzodiazepines | No restraint |
| GnRH analogs | No restraint |
| Tamoxifen | No restraint |
| Tamoxifen or GnRH analogs or aromatase inhibitors exposure | No restraint |

GnRH, gonadotropin-releasing hormone.

**Supplementary Table 3: Menopause descriptor concept IDs**

| **Concept name** | **Vocabulary** | **Concept ID** |
| --- | --- | --- |
| Postmenopausal osteopenia | SNOMED | 735614005 |
| Postmenopausal frontal fibrosing alopecia | SNOMED | 403325003 |
| Perimenopausal atrophic vaginitis | SNOMED | 408386002 |
| Postmenopausal postcoital bleeding | SNOMED | 415149004 |
| Menopausal headache | SNOMED | 198438009 |
| Menopausal flushing | SNOMED | 198436008 |
| Menopausal sleeplessness | SNOMED | 198437004 |
| Menopausal concentration lack | SNOMED | 198439001 |
| Menopausal symptom | SNOMED | 21801002 |
| Postmenopausal osteoporosis with pathological fracture | SNOMED | 203453001 |
| Menopause symptoms present | SNOMED | 170951000 |
| H/O: postmenopausal bleeding | SNOMED | 161788002 |
| Postmenopausal urethral atrophy | SNOMED | 21237001 |
| Postmenopausal osteoporosis | SNOMED | 102447009 |
| Postmenopausal bleeding | SNOMED | 76742009 |
| Climacteric arthritis | SNOMED | 77888002 |
| Menopausal osteoporosis | SNOMED | 32369003 |
| Menopausal depression | SNOMED | 84788008 |
| Disorder associated with menstruation AND/OR menopause | SNOMED | 106002000 |
| Dermatosis of menopause | SNOMED | 402804002 |
| Genitourinary syndrome of menopause | SNOMED | 16779841000119104 |
| Climacteric arthritis of the pelvic region and thigh | SNOMED | 201981006 |
| Climacteric arthritis of the ankle and/or foot | SNOMED | 201983009 |
| Climacteric arthritis of multiple sites | SNOMED | 201985002 |
| Climacteric arthritis of the hand | SNOMED | 201980007 |
| Climacteric arthritis of the shoulder region | SNOMED | 201977006 |
| Climacteric flushing | SNOMED | 427368007 |
| Climacteric arthritis of spine | SNOMED | 429298008 |
| Postmenopausal flushing | SNOMED | 403389006 |

H/O, history of.

Supplementary Table 4: Symptoms without menopause descriptor concept IDs

| **Concept Name** | **Vocabulary** | **Code** |
| --- | --- | --- |
| Night sweats | SNOMED | 42984000 |
| Hot sweats | SNOMED | 224962007 |
| Abnormal vasomotor function | SNOMED | 70670009 |
| On melatonin for sleep disorder | SNOMED | 887900000000000 |
| Disruptions of 24 hour sleep-wake cycle | SNOMED | 31490000000000 |
| Adjustment insomnia | SNOMED | 472819006 |
| Daytime somnolence | SNOMED | 141000000000 |
| Does not sleep without sedation | SNOMED | 1069000000000000 |
| Does not sleep with sedation | SNOMED | 1073000000000000 |
| Does not sleep | SNOMED | 1073000000000000 |
| Difficulty sleeping without sedation | SNOMED | 1071000000000000 |
| Difficulty sleeping with sedation | SNOMED | 1071000000000000 |
| Acute insomnia | SNOMED | 762348004 |
| Idiopathic hypersomnia associated with long sleep time | SNOMED | 442416002 |
| Long sleeper syndrome | SNOMED | 443664006 |
| Idiopathic hypersomnia without long sleep time | SNOMED | 442292004 |
| Periodic leg movements of sleep | SNOMED | 445140006 |
| Sleep dysfunction with sleep stage disturbance | SNOMED | 441877007 |
| Insomnia co-occurrent and due to nocturnal myoclonus | SNOMED | 93680000000000 |
| Not easily wakened from sleep | SNOMED | 733504007 |
| Chronic insomnia | SNOMED | 724748004 |
| Sleep related movement disorder | SNOMED | 84990000000000 |
| Sleep related rhythmic movement disorder | SNOMED | 430893009 |
| Periodic limb movement disorder | SNOMED | 418763003 |
| Middle insomnia | SNOMED | 67233009 |
| Initial insomnia | SNOMED | 59050008 |
| Sleep terror disorder | SNOMED | 89675003 |
| Delayed onset of sleep | SNOMED | 401161007 |
| Poor sleep pattern | SNOMED | 314938000 |
| Intermittent drowsiness | SNOMED | 315243006 |
| Excessive somnolence | SNOMED | 372947007 |
| Sleep-wake schedule disorder, frequently changing type | SNOMED | 54532007 |
| Mixed insomnia | SNOMED | 54230003 |
| Sleep-related groaning | SNOMED | 429266002 |
| Nightmares | SNOMED | 419145002 |
| C/O - somnolence | SNOMED | 272026007 |
| Complaining of insomnia | SNOMED | 272025006 |
| Drowsy | SNOMED | 271782001 |
| Gets drowsiness | SNOMED | 267088000 |
| Hypersomnia disorder related to menstruation | SNOMED | 426943005 |
| Sleep attack | SNOMED | 427426006 |
| Psychophysiologic insomnia | SNOMED | 425832009 |
| Non-organic parasomnia | SNOMED | 425476007 |
| Difficulty sleeping | SNOMED | 301345002 |
| Reversed sleep-wake cycle | SNOMED | 192008004 |
| Repeated rapid eye movement sleep interruptions | SNOMED | 192004002 |
| Nonorganic insomnia | SNOMED | 192454004 |
| Symptoms interfere with sleep | SNOMED | 248259004 |
| Circumstances interfere with sleep | SNOMED | 248258007 |
| Not getting enough sleep | SNOMED | 248256006 |
| Oversleeps | SNOMED | 248261008 |
| Always sleepy | SNOMED | 248262001 |
| Unrefreshed by sleep | SNOMED | 248260009 |
| Sleep automatism | SNOMED | 247962006 |
| Pavor diurnus | SNOMED | 17402007 |
| Hypersomnia of non-organic origin | SNOMED | 230488004 |
| Excessive daytime sleepiness - normal night sleep | SNOMED | 230489007 |
| Excessive daytime sleepiness with sleep paralysis | SNOMED | 230492006 |
| Excessive day and night-time sleepiness | SNOMED | 230490003 |
| O/E - drowsy | SNOMED | 162704004 |
| Late insomnia | SNOMED | 162204000 |
| C/O nightmares | SNOMED | 162207007 |
| Insomnia due to anxiety and fear | SNOMED | 436000000000000 |
| Recurrent hypersomnia | SNOMED | 426451004 |
| Sleep-wake schedule disorder, advanced phase type | SNOMED | 31537005 |
| Non-24 hour sleep-wake cycle | SNOMED | 230496009 |
| Parasomnia | SNOMED | 58690002 |
| Persistent insomnia | SNOMED | 191997003 |
| Disorders of initiating and maintaining sleep | SNOMED | 194437008 |
| REM sleep behavior disorder | SNOMED | 415238003 |
| Hypersomnia | SNOMED | 77692006 |
| Insomnia | SNOMED | 193462001 |
| Irregular sleep-wake pattern | SNOMED | 271793004 |
| Disorder of sleep-wake cycle | SNOMED | 271794005 |
| Dyssomnia | SNOMED | 44186003 |
| Sleep disorder | SNOMED | 39898005 |
| Transient insomnia | SNOMED | 268652009 |
| Sleep-wake schedule disorder, delayed phase type | SNOMED | 80623000 |
| Sleep related bruxism | SNOMED | 274950005 |
| Sleep walking disorder | SNOMED | 80495009 |
| Non-organic disorder of the sleep-wake schedule | SNOMED | 268722008 |
| Non-organic sleep disorder | SNOMED | 270487001 |
| Restless legs | SNOMED | 32914008 |
| Repetitive intrusions of sleep | SNOMED | 268654005 |
| Sleep paralysis | SNOMED | 277180005 |
| Prolonged sleep | SNOMED | 48091006 |
| Cramp in lower limb associated with sleep | SNOMED | 431800000000000 |
| Assessment using Sleep Inertia Questionnaire | SNOMED | 865920004 |
| Restless sleep | SNOMED | 12262002 |
| Not easily wakened from sleep walking | SNOMED | 247960003 |
| Low level of awareness while sleep walking | SNOMED | 247955002 |
| Low level of reactivity while sleep walking | SNOMED | 247956001 |
| Unresponsive to communication while sleep walking | SNOMED | 247959008 |
| Low level of motor skill while sleep walking | SNOMED | 247957005 |
| No recollection of sleep walk | SNOMED | 247961004 |
| Keeps falling asleep | SNOMED | 248219002 |
| Cannot sleep at all | SNOMED | 248255005 |
| Small sharp transients during sleep | SNOMED | 251453002 |
| Sleep drunkenness | SNOMED | 192003008 |
| Light sleep | SNOMED | 29373008 |
| Sleep pattern disturbance | SNOMED | 26677001 |
| Sleep state misperception | SNOMED | 427745001 |
| Disturbance in sleep behavior | SNOMED | 53888004 |
| Temporal periods relating to sleep | SNOMED | 309609009 |
| Phototherapy: sleep regulation | SNOMED | 406175001 |
| Sleep restriction therapy | SNOMED | 440089000 |
| Sleeptalking | SNOMED | 36321004 |
| Terminal insomnia | SNOMED | 67062000 |
| Abnormal rapid eye movement sleep | SNOMED | 69020003 |
| Finding related to sleep walking | SNOMED | 419057008 |
| Sleep deprivation | SNOMED | 130989002 |
| Insufficient rest/sleep for physical condition | SNOMED | 422730001 |
| Insufficient rest/sleep for age | SNOMED | 425202006 |
| Pain onset during sleep | SNOMED | 429038000 |
| Sleep-related dissociative disorder | SNOMED | 429571005 |
| Assessment using Pittsburgh Sleep Quality Index | SNOMED | 763105008 |
| Somnambulism co-occurrent with sleep terror disorder | SNOMED | 23870000000000 |
| Sleeps during day | SNOMED | 713567005 |
| Propriospinal myoclonus at sleep onset | SNOMED | 789010008 |
| Idiopathic sleep related non-obstructive alveolar hypoventilation | SNOMED | 441910000 |
| Sleep related hallucinations | SNOMED | 445112001 |
| Recurrent isolated sleep paralysis | SNOMED | 442120007 |
| Sleep dysfunction with arousal disturbance | SNOMED | 442176004 |
| Pittsburgh Sleep Quality Index score | SNOMED | 761879005 |
| Does sleep with sedation | SNOMED | 1074000000000000 |
| Unable to sleep with sedation | SNOMED | 1076000000000000 |
| Unable to sleep without sedation | SNOMED | 1076000000000000 |
| Able to sleep with sedation | SNOMED | 799000000000000 |
| Monitoring of insomnia | SNOMED | 1157000000 |
| Insomnia medication review | SNOMED | 452800000000000 |
| Education about insomnia | SNOMED | 452900000000000 |
| Cognitive behavioral therapy for insomnia | SNOMED | 868185009 |
| Terminal mood insomnia | SNOMED | 3277001 |
| Middle mood insomnia | SNOMED | 45291004 |
| Mood insomnia | SNOMED | 53758003 |
| Education about stimulus control behavior in insomnia | SNOMED | 440313002 |
| Primary insomnia | SNOMED | 3972004 |
| Rebound insomnia | SNOMED | 88982005 |
| Initial mood insomnia | SNOMED | 38376001 |
| ISI (Insomnia Severity Index) score | SNOMED | 762989006 |
| Signposting for digital cognitive behavioural therapy for insomnia | SNOMED | 1107000000000000 |
| Referral for digital cognitive behavioural therapy for insomnia | SNOMED | 1107000000000000 |
| Self referral for digital cognitive behavioural therapy for insomnia | SNOMED | 1107000000000000 |
| Digital cognitive behavioural therapy for insomnia | SNOMED | 111000000000000 |
| Assessment using Insomnia Severity Index | SNOMED | 761885003 |
| Persistent hypersomnia | SNOMED | 191999000 |
| Transient hypersomnia | SNOMED | 268653004 |
| Mood hypersomnia | SNOMED | 59445005 |
| Primary hypersomnia | SNOMED | 36124002 |
| Behaviorally induced hypersomnia | SNOMED | 724749007 |
| Primary hyposomnia | SNOMED | 105500000000000 |
| Daytime hypersomnia | SNOMED | 31770000000000 |
| Idiopathic hypersomnia | SNOMED | 3731000000000 |
| Abnormal circadian rhythm | SNOMED | 15976004 |
| Wakefulness finding | SNOMED | 365930002 |
| Wakefulness | SNOMED | 27625002 |
| Dream anxiety disorder | SNOMED | 111487009 |
| Psychogenic fatigue | SNOMED | 442099003 |
| Occasionally tired | SNOMED | 713568000 |
| Fatigue | SNOMED | 84229001 |
| Tired all the time | SNOMED | 267032009 |
| Attacks of weakness | SNOMED | 248278004 |
| Tired on least exertion | SNOMED | 248269005 |
| Tired | SNOMED | 224960004 |
| Malaise and fatigue | SNOMED | 271795006 |
| Asthenia | SNOMED | 13791008 |
| Level of fatigue | SNOMED | 440398005 |
| Management of fatigue | SNOMED | 713121000 |
| Senile asthenia | SNOMED | 18726006 |
| Lethargy | SNOMED | 214264003 |
| Anxiety about lethargy | SNOMED | 323300000000000 |
| Palpitations | SNOMED | 80313002 |
| Intermittent palpitations | SNOMED | 102590007 |
| Fluttering heart | SNOMED | 161969004 |
| Pounding heart | SNOMED | 248657009 |
| Palpitations - rapid | SNOMED | 248648003 |
| Palpitations with regular rhythm | SNOMED | 428919002 |
| Irregular palpitations | SNOMED | 240200000000000 |
| Palpitations - regular | SNOMED | 240200000000000 |
| Palpitations care pathway | SNOMED | 907800000000000 |
| Exocardial pulsation associated with heart beat | SNOMED | 248662005 |
| Awareness of heart beat | SNOMED | 271820000 |
| Finding related to awareness of heart beat | SNOMED | 366182005 |
| Awareness of heart beat | SNOMED | 277766008 |
| Joint pain | SNOMED | 57676002 |
| Shoulder joint pain | SNOMED | 267949000 |
| Hand joint pain | SNOMED | 202472008 |
| Multiple joint pain | SNOMED | 35678005 |
| Pain of right knee joint | SNOMED | 468200000000000 |
| Pain of left knee joint | SNOMED | 468300000000000 |
| Pain of joint of knee | SNOMED | 1004000000 |
| Joint pain in right hand | SNOMED | 1077000000000000 |
| Joint pain in left hand | SNOMED | 1077000000000000 |
| Pain of joint of right foot | SNOMED | 1077000000000000 |
| Pain of left temporomandibular joint | SNOMED | 12240000000000000 |
| Pain of right temporomandibular joint | SNOMED | 12240000000000000 |
| Pain in left sacroiliac joint | SNOMED | 12240000000000000 |
| Pain in right sacroiliac joint | SNOMED | 12240000000000000 |
| Bilateral hip joint pain | SNOMED | 12250000000000000 |
| Bilateral ankle joint pain | SNOMED | 12250000000000000 |
| Bilateral shoulder joint pain | SNOMED | 12250000000000000 |
| Pain of joint of right lower leg | SNOMED | 15740000000000000 |
| Pain of joint of left lower leg | SNOMED | 15740000000000000 |
| Pain of right ankle joint | SNOMED | 15920000000000000 |
| Pain of left shoulder joint | SNOMED | 15920000000000000 |
| Pain of right shoulder joint | SNOMED | 15920000000000000 |
| Pain of left elbow joint | SNOMED | 15920000000000000 |
| Pain of right elbow joint | SNOMED | 15920000000000000 |
| Pain of left hip joint | SNOMED | 31680000000000000 |
| Pain of left ankle joint | SNOMED | 15920000000000000 |
| Bilateral sacroiliac joint pain | SNOMED | 15630000000000000 |
| Pain of joint of bilateral lower legs | SNOMED | 15740000000000000 |
| Pain of joint of left foot | SNOMED | 10770000000000000 |
| Bilateral elbow joint pain | SNOMED | 12250000000000000 |
| Pain in right hip joint | SNOMED | 316900000000000 |
| Facet joint pain | SNOMED | 247369005 |
| Distal radioulnar joint pain | SNOMED | 202481002 |
| Wrist joint pain | SNOMED | 202482009 |
| Sacroiliac joint pain | SNOMED | 202487003 |
| Subtalar joint pain | SNOMED | 202491008 |
| Sternoclavicular joint pain | SNOMED | 202478007 |
| Acromioclavicular joint pain | SNOMED | 202479004 |
| Elbow joint pain | SNOMED | 202480001 |
| Metacarpophalangeal joint pain | SNOMED | 202483004 |
| Ankle joint pain | SNOMED | 202490009 |
| Talonavicular joint pain | SNOMED | 202493006 |
| Lesser metatarsophalangeal joint pain | SNOMED | 202496003 |
| Proximal interphalangeal joint of finger pain | SNOMED | 202484005 |
| Distal interphalangeal joint of finger pain | SNOMED | 202485006 |
| First metatarsophalangeal joint pain | SNOMED | 202495004 |
| Tibiofibular joint pain | SNOMED | 202489000 |
| Interphalangeal joint of toe pain | SNOMED | 202497007 |
| Lumbar facet joint pain | SNOMED | 279063004 |
| Foot joint pain | SNOMED | 279066007 |
| Metatarsophalangeal joint pain | SNOMED | 279067003 |
| Hip pain | SNOMED | 49218002 |
| Pain on passive stretch of joint | SNOMED | 429531000 |
| Cervical facet joint pain | SNOMED | 298253002 |
| Thoracic facet joint pain | SNOMED | 298254008 |
| Pain on joint movement | SNOMED | 298255009 |
| Pain of right acromioclavicular joint | SNOMED | 774136009 |
| Pain of left acromioclavicular joint | SNOMED | 774137000 |
| Vertebral joint pain | SNOMED | 87380000000000 |
| Pain of right sternoclavicular joint | SNOMED | 1076000000000000 |
| Pain of left sternoclavicular joint | SNOMED | 1076000000000000 |
| Bilateral acromioclavicular joint pain | SNOMED | 15640000000000000 |
| Bilateral foot joint pain | SNOMED | 12250000000000000 |
| Joint pain of pelvic region | SNOMED | 713413001 |
| Chronic sacroiliac joint pain | SNOMED | 782661001 |
| Bilateral pain of joint of hands | SNOMED | 1690000000000000 |
| Bilateral temporomandibular joint pain | SNOMED | 15640000000000000 |
| Pain on stress testing of joint | SNOMED | 298400000000000 |
| Acute pain of joint of knee | SNOMED | 112600000000000 |
| Dyspareunia | SNOMED | 71315007 |
| Psychologic dyspareunia | SNOMED | 41021005 |
| Superficial pain on intercourse | SNOMED | 247418006 |
| Deep pain on intercourse | SNOMED | 247419003 |
| Dyspareunia due to non-psychogenic cause in the female | SNOMED | 198402002 |
| Non-psychogenic dyspareunia | SNOMED | 286992006 |
| Vulval superficial dyspareunia | SNOMED | 313344000 |
| Female deep pain on intercourse | SNOMED | 16690000000000000 |
| Pain in female genitalia on intercourse | SNOMED | 81712001 |
| Pain following sexual intercourse | SNOMED | 423893004 |
| Vaginal dryness on intercourse | SNOMED | 248766002 |
| Vaginal dryness | SNOMED | 31908003 |
| Atrophic vaginitis | SNOMED | 52441000 |
| Atrophy of vagina | SNOMED | 297147009 |
| Atrophic vulva | SNOMED | 248861000 |
| Finding of frequency of urination | SNOMED | 300471006 |
| Increased frequency of urination | SNOMED | 162116003 |
| Frequency of urination | SNOMED | 364198000 |
| Urgent desire to urinate | SNOMED | 75088002 |
| Micturition frequency and polyuria | SNOMED | 274734008 |
| Polyuria | SNOMED | 28442001 |
| Nocturnal polyuria | SNOMED | 1099000000000000 |
| Nocturia | SNOMED | 139394000 |
| Frequency of nocturia | SNOMED | 203200000000000 |
| Urge incontinence of urine | SNOMED | 87557004 |
| Genuine stress incontinence | SNOMED | 22220005 |
| Nocturnal enuresis | SNOMED | 8009008 |
| Female stress incontinence | SNOMED | 60241006 |
| Unaware of passing urine | SNOMED | 249284009 |
| Overflow incontinence of urine | SNOMED | 397878005 |
| Urinary incontinence | SNOMED | 165232002 |
| Neurogenic incontinence | SNOMED | 386710007 |
| Mixed urinary incontinence | SNOMED | 413343005 |
| Extraurethral urinary incontinence | SNOMED | 1145000000 |
| Primary stress incontinence | SNOMED | 1149000000 |
| Giggle incontinence of urine | SNOMED | 236660009 |
| Orgasmic incontinence of urine | SNOMED | 236663006 |
| Psychogenic urinary incontinence | SNOMED | 236667007 |
| Total urinary incontinence | SNOMED | 129853007 |
| Postural urinary incontinence | SNOMED | 236665004 |
| Post-micturition incontinence | SNOMED | 236664000 |
| Cough - urge incontinence of urine | SNOMED | 236659004 |
| Urinary incontinence of non-organic origin | SNOMED | 42112009 |
| Number of urinary incontinence episodes | SNOMED | 422058003 |
| Reflex incontinence of urine | SNOMED | 90987003 |
| Urinary incontinence care management | SNOMED | 408979003 |
| Urinary incontinence care assessment | SNOMED | 408977001 |
| Urinary incontinence care education | SNOMED | 408978006 |
| Urinary incontinence care: enuresis | SNOMED | 386489000 |
| Urinary incontinence care | SNOMED | 386488008 |
| Double incontinence | SNOMED | 78459008 |
| Daily urinary incontinence | SNOMED | 461200000000000 |
| Incontinence without sensory awareness | SNOMED | 448521006 |
| Intermittent urinary incontinence | SNOMED | 450841000 |
| Number of urinary incontinence episodes in 24 hours | SNOMED | 816300000000000 |
| Sneezing incontinence of urine | SNOMED | 840800000000000 |
| Overactive bladder | SNOMED | 786457000 |
| Dry skin | SNOMED | 16386004 |
| Dry skin dermatitis | SNOMED | 260046006 |
| Dry skin of abdomen | SNOMED | 827180003 |
| Severe dry skin | SNOMED | 702757002 |
| Conjunctival xerosis | SNOMED | 64718006 |
| Bilateral conjunctival xerosis of eyes | SNOMED | 346600000000000 |
| Senile xeroderma | SNOMED | 238597002 |
| Dry cornea | SNOMED | 425512003 |
| Asteatosis cutis | SNOMED | 89105000 |
| Xeroderma of eyelid | SNOMED | 55846006 |
| Xeroderma of right upper eyelid | SNOMED | 330800000000000 |
| Xeroderma of right eyelid | SNOMED | 330800000000000 |
| Xeroderma of left upper eyelid | SNOMED | 330900000000000 |
| Xeroderma of left eyelid | SNOMED | 330900000000000 |
| Dry skin dermatitis | SNOMED | 260046006 |
| Xeroderma | SNOMED | 52475004 |
| Xeroderma of lower eyelid | SNOMED | 700298002 |
| Xeroderma of upper eyelid | SNOMED | 700346002 |
| Keratoconjunctivitis sicca (excluding SjÃ¶gren syndrome) | SNOMED | 193776001 |
| Keratoconjunctivitis sicca | SNOMED | 302896008 |
| Tear film insufficiency | SNOMED | 46152009 |
| Dry eye syndrome of right eye | SNOMED | 335300000000000 |
| Dry eye syndrome of left eye | SNOMED | 34100000000000 |
| Dry eyes | SNOMED | 162290004 |
| Anxiety | SNOMED | 48694002 |
| Anxious character | SNOMED | 286710008 |
| Worried | SNOMED | 79015004 |
| Current View Provisional Problem Description item 3 score - anxious generally | SNOMED | 987300000000000 |
| Anxiety | SNOMED | 48694002 |
| Anxiety neurosis | SNOMED | 207363009 |
| Acknowledging anxiety | SNOMED | 225216003 |
| Anxiety and fear | SNOMED | 247805009 |
| Recurrent anxiety | SNOMED | 191709001 |
| Anxiety state | SNOMED | 198288003 |
| Anxiety attack | SNOMED | 300895004 |
| Level of anxiety | SNOMED | 286644009 |
| Severe anxiety (panic) | SNOMED | 80583007 |
| Free-floating anxiety | SNOMED | 81350009 |
| Level of anxiety | SNOMED | 405051006 |
| Moderate anxiety | SNOMED | 61387006 |
| Anxiety hyperventilation | SNOMED | 69479009 |
| Mild anxiety | SNOMED | 70997004 |
| Anxiety hysteria | SNOMED | 231506008 |
| Generalized anxiety disorder | SNOMED | 21897009 |
| Volatile Mood | SNOMED | 225657003 |
| Unpredictable in mood | SNOMED | 225656007 |
| Diminished libido due to mood | SNOMED | 14661003 |
| Indifference | SNOMED | 20602000 |
| Non-delusional perplexed mood | SNOMED | 276245005 |
| Mood swings | SNOMED | 18963009 |
| Diurnal variation of mood | SNOMED | 247800004 |
| Hypomanic mood | SNOMED | 281257007 |
| Variability of mood | SNOMED | 281129008 |
| Rebound mood swings | SNOMED | 191664002 |
| Cyclic mood swings | SNOMED | 307076007 |
| Dysphoric mood | SNOMED | 30819006 |
| Crying associated with mood | SNOMED | 271951008 |
| Disturbance in mood | SNOMED | 48079002 |
| Emotional hypersensitivity | SNOMED | 421369008 |
| Fearful mood | SNOMED | 367545001 |
| Physiological disturbance associated with mood | SNOMED | 71529007 |
| Depressed mood | SNOMED | 366979004 |
| Anxiety about mood | SNOMED | 323400000000000 |
| HoNOS (Health of the Nation Outcome Scales) for working age adults rating scale 7 score - problems with depressed mood | SNOMED | 979700000000000 |
| Current View Provisional Problem Description item 11 score - extremes of mood | SNOMED | 987400000000000 |
| Current View Provisional Problem Description item 9 score - depression/low mood | SNOMED | 987300000000000 |
| Emotional state finding | SNOMED | 106126000 |
| Physical AND emotional exhaustion state | SNOMED | 58535001 |
| Emotional state characteristics | SNOMED | 363872004 |
| Management of negative emotional state | SNOMED | 710965006 |
| Irritability and anger | SNOMED | 274646000 |
| Feeling irritable | SNOMED | 55929007 |
| Demoralization | SNOMED | 45763006 |
| Indifference | SNOMED | 20602000 |
| Feeling agitated | SNOMED | 24199005 |
| Restlessness and agitation | SNOMED | 274647009 |
| Level of agitation | SNOMED | 440651009 |
| Feeling unhappy | SNOMED | 420038007 |

Supplementary Table 5: Natural menopause diagnosis concept IDs

| **Concept name** | **Vocabulary** | **Concept ID** | **Diagnosis** | **Possible** | | **Probable** |
| --- | --- | --- | --- | --- | --- | --- |
| Luteinizing hormone and follicle-stimulating hormone check | SNOMED | 709262003 | Yes | | Yes | No |
| Perimenopausal state | SNOMED | 161541000119104 | Yes | | Yes | No |
| Anovulatory amenorrhea | SNOMED | 444769001 | Yes | | Yes | No |
| Secondary amenorrhea | SNOMED | 156036003 | Yes | | Yes | No |
| Premature ovarian failure due to autoimmune oophoritis | SNOMED | 721198006 | Yes | | Yes | No |
| Sclerosing dysplasia of bone, ichthyosis, premature ovarian failure syndrome | SNOMED | 722114007 | Yes | | Yes | No |
| Idiopathic premature ovarian failure | SNOMED | 721176002 | Yes | | Yes | No |
| Secondary physiologic amenorrhea | SNOMED | 86030004 | Yes | | Yes | Yes |
| Difficulty managing menopause | SNOMED | 423478005 | Yes | | Yes | Yes |
| Postmenopausal state | SNOMED | 76498008 | Yes | | Yes | Yes |
| Menopausal problem | SNOMED | 68811000 | Yes | | Yes | Yes |
| Primary ovarian failure | SNOMED | 65846009 | Yes | | Yes | No |
| Physiologic amenorrhea | SNOMED | 44811000 | Yes | | Yes | No |
| Resistant ovary syndrome | SNOMED | 80956002 | Yes | | Yes | No |
| Menopause education | SNOMED | 313207002 | Yes | | Yes | Yes |
| Amenorrhea associated with obesity | SNOMED | 413487000 | Yes | | Yes | No |
| Menopause finding | SNOMED | 276477006 | Yes | | Yes | Yes |
| Secondary oligomenorrhea | SNOMED | 34272008 | Yes | | Yes | No |
| Perimenopausal disorder | SNOMED | 266607004 | Yes | | Yes | No |
| Premenopausal amenorrhea | SNOMED | 32590007 | Yes | | Yes | No |
| Menopause ovarian failure | SNOMED | 237138004 | Yes | | Yes | Yes |
| Menopause present | SNOMED | 289903006 | Yes | | Yes | Yes |
| Female climacteric state | SNOMED | 198435007 | Yes | | Yes | Yes |
| Menopause monitoring status | SNOMED | 243875009 | Yes | | Yes | Yes |
| Delayed menopause | SNOMED | 20045006 | Yes | | Yes | Yes |
| Menopause sexual education | SNOMED | 170962000 | Yes | | Yes | Yes |
| Menopause: bone density check | SNOMED | 170953002 | Yes | | Yes | Yes |
| Menopause initial assessment | SNOMED | 170949004 | Yes | | Yes | Yes |
| Menopause dietary education | SNOMED | 170961007 | Yes | | Yes | Yes |
| Menopause follow-up assessment | SNOMED | 170950004 | Yes | | Yes | Yes |
| Menopause | SNOMED | 161712005 | Yes | | Yes | Yes |
| Incipient ovarian failure | SNOMED | 237137009 | Yes | | Yes | No |
| Normal menopause | SNOMED | 237123000 | Yes | | Yes | Yes |
| Autoimmune primary ovarian failure | SNOMED | 237790001 | Yes | | Yes | No |
| Premature ovarian failure | SNOMED | 237788002 | Yes | | Yes | No |
| Menopausal and postmenopausal disorders | SNOMED | 266677000 | Yes | | Yes | Yes |
| Menopausal syndrome | SNOMED | 123756000 | Yes | | Yes | Yes |
| Premature menopause | SNOMED | 373717006 | Yes | | Yes | Yes |
| Abnormal presence of endometrial cells in specimen from a menopausal woman | SNOMED | 103610009 | Yes | | Yes | Yes |
| FH: Early menopause | SNOMED | 160397006 | Yes | | Yes | Yes |
| FH: Late menopause | SNOMED | 160398001 | Yes | | Yes | Yes |
| During menopause | SNOMED | 303111005 | Yes | | Yes | Yes |
| After menopause | SNOMED | 307429007 | Yes | | Yes | Yes |
| Menopause monitoring | SNOMED | 268530007 | Yes | | Yes | Yes |
| History of normal menopause | SNOMED | 427956008 | Yes | | Yes | Yes |
| Counseling for menopause | SNOMED | 439421000 | Yes | | Yes | Yes |
| Temporal periods relating to menopause | SNOMED | 309605003 | Yes | | Yes | No |
| Age at menopause | SNOMED | 773251000 | Yes | | Yes | Yes |
| Oligomenorrhea | SNOMED | 52073004 | Yes | | Yes | No |
| Amenorrhea | SNOMED | 14302001 | Yes | | Yes | No |
| H/O: amenorrhea | SNOMED | 161780009 | Yes | | Yes | No |
| Pathologic amenorrhea | SNOMED | 56561006 | Yes | | Yes | No |
| Ovarian failure | SNOMED | 111550004 | Yes | | Yes | No |
| Secondary ovarian failure | SNOMED | 267400004 | Yes | | Yes | No |

FH, family history; H/O, history of.

**Supplementary Table 6: Codes used to identify VMS-related records**

| **Type** | **Concept Name** | **Vocabulary** | **Code** | **Possible** | **Probable** |
| --- | --- | --- | --- | --- | --- |
| Original | Postmenopausal flushing | SNOMED | 403389006 | Yes | Yes |
| Original | Night sweats | SNOMED | 42984000 | Yes | Yes |
| Original | Climacteric flushing | SNOMED | 427368007 | Yes | Yes |
| Original | Menopausal flushing | SNOMED | 198436008 | Yes | Yes |
| Original | Hot sweats | SNOMED | 224962007 | Yes | Yes |
| Original | Menopausal and postmenopausal disorders | SNOMED | 266677000 | Yes | No |
| Original | Menopausal syndrome | SNOMED | 123756000 | Yes | No |
| Athena | Abnormal vasomotor function | SNOMED | 70670009 | Yes | No |
| Athena | Disorder associated with menstruation AND/OR menopause | SNOMED | 106002000 | Yes | No |
| Athena | Menopause symptoms present | SNOMED | 170951000 | Yes | No |
| Athena | Menopausal symptom | SNOMED | 21801002 | Yes | No |
| Athena | Menopausal problem | SNOMED | 68811000 | Yes | No |

Supplementary Figure 1: Age distribution of menopause cohorts

***C1:*** women of natural menopausal age (aged 40–65); ***C2a:*** women aged 40–65 with a possible menopause diagnosis; ***C2b:*** women aged 40–65 with a possible menopause diagnosis and no prior HT or non-HT treatment; ***C2c:*** women aged 40–65 with a probable menopause diagnosis; ***C2d:*** women aged 40–65 with a probable menopause diagnosis and no prior HT or non-HT treatment; ***C3a:*** women aged 40–65 with a possible menopause diagnosis or symptom with or without menopause descriptor; ***C3b:*** women aged 40–65 with a possible menopause diagnosis or symptom with or without menopause descriptor and no prior HT or non-HT treatment; ***C3c:*** women aged 40–65 with a probable menopause diagnosis or symptom with menopause descriptor; ***C2d:*** women aged 40–65 with a probable menopause diagnosis or symptom with menopause descriptor and no prior HT or non-HT treatment; ***C4a:*** women aged 40–65 with a possible menopause diagnosis or symptom with or without menopause descriptor or a HT/non-HT treatment; ***C4b:*** women aged 40–65 with a probable menopause diagnosis or symptom with menopause descriptor or a HT/non-HT treatment; ***C5:*** women aged 40–65 with a HT/non-HT treatment. HT, hormone therapy; UK, United Kingdom; US, United States.
